# Playing With Fire: the Effects of Institutional Dysfunctions in Postdisaster Business Recovery

**DOI:** 10.1111/risa.70085

**Published:** 2025-07-29

**Authors:** Joseph Amankwah‐Amoah, Yaw A. Debrah

**Affiliations:** ^1^ Durham University Business School, Durham University Durham UK; ^2^ Independent Researcher & Emeritus Professor of Management Swansea UK

**Keywords:** Africa, failure, fire, hazards, institutional dysfunction, postdisaster recovery, risks

## Abstract

Although previous research has illuminated our scholarly understanding of the general effects of institutional dysfunction, there remains a conspicuous dearth of research on how institutional dysfunction shapes postdisaster business recovery. Drawing on insights from entrepreneurs affected by fire outbreaks in marketplaces in a developing economy, this study uncovers two unique and interrelated stages that illuminate how institutional dysfunction manifests over time. Throughout these stages, we observe institutional dysfunction acting as an “accelerator” in the wake of the fire outbreaks, ultimately leading to business closures. Intriguingly, the study reveals that dysfunctions not only expose the faulty practices and routines of businesses but also highlight the fragilities and obstructive nature of existing formal and informal institutions. This multifaceted analysis unravels the more intricate process of how the effects of institutional dysfunction unfold over time, commencing with the sensemaking of marketplace fire outbreaks and focusing on institutional shortcomings and inadequacies (i.e., Stage 1). The unfulfilled promised financial and nonfinancial support by various political actors culminate in a downward spiral, ultimately resulting in disaster‐induced business demise (i.e., Stage 2). The theoretical implications and practical risk mitigation strategies of the study are outlined.

## 1 Introduction

In the past two decades, there has been a growing body of research on natural and man‐made disasters, such as heatwaves, fire outbreaks, hurricanes, earthquakes, and epidemics, along with studies shedding light on their effects, including environmental pollution, population displacement, and infrastructure damage in the global south (Marshall et al. 2020; Nielsen et al. 2023; Peek and Guikema 2021; World Bank 2013). In recent years, the number of disaster events and their frequency has increased exponentially, with a threefold increase over the last fifty years, leading to a diverse range of adverse impacts on communities and businesses (Marshall et al. 2020; Nielsen et al. 2023; World Bank 2013). Typical developing economies are characterized by institutional dysfunctions, such as high informality in the economy, underdeveloped transport infrastructure, poor hygiene and sanitation, and persistent incidents of fire, which can impede business activities (Asante and Helbrecht 2020; Bromley and Mackie 2009).

Although institutional dysfunction is a pervasive force shaping the business atmosphere in developing economies (Amankwah‐Amoah et al. 2018; Barnard and Mamabolo 2022; Rodgers et al. 2022), as well as a quintessential determinant of firm behavior (Peng 2002; Shrestha et al. 2010), the potential mechanisms through which institutional dysfunction shapes postdisaster business recovery remain underexplored. While a growing body of research has illuminated scholarly insights into the effects of institutional dysfunction (e.g., Barnard and Mamabolo 2022) and postdisaster business recovery (e.g., Morrish and Jones 2020), the potential linkages between the two remain largely unexplored. Despite a growing body of research on both topics, unfortunately, the current literature is disorganized, disjointed, and fails to explain the potential linkages between the two. Surprisingly limited scholarly attention has been paid to how institutional dysfunction shapes postdisaster recovery efforts. Against this backdrop, the primary purpose of this study is to examine how institutional dysfunction shapes postdisaster business recovery.

A marketplace fire outbreak is a multifaceted hazardous event epitomized by accidental combustion within a market trading area or space (Abunyewah et al. 2023; Ageboba et al. 2023; Oteng‐Ababio et al. 2015). In other words, it involves a blaze engulfing part or the entire major trading hub for goods and services. This disruptive event typically encompasses the rapid or incremental spread of flames within a market trading center or commercial areas, leading to damage to infrastructure, goods, lives, and property (Abunyewah et al. 2023; Nyame‐Asiamah et al. 2023; Oteng‐Ababio and Sarpong 2015). In some countries, marketplaces are also cultural centers, a source of vitality for urban life, and signal centers for demonstrating and celebrating the shared cultural heritage of the people (Abunyewah et al. 2023). Accompanying major fire events often have significant detrimental effects on both public and private property in commercial places (Oteng‐Ababio et al. 2015; World Bank 2013).

The paper makes several contributions to the literature. First, although previous research has illuminated the general effects of institutional dysfunction (e.g. Barnard and Mamabolo 2022; Ofori‐Dankwa and Julian 2013), there remains a conspicuous dearth of scholarly works on the effects in the aftermath of a disaster. Building upon the seminal work on institutions (North 1990; Peng et al. 2023; Scott 2013), this study delves into informal and formal institutional inefficiencies that enhance business vulnerabilities. Thus, the study illuminates the intricate interplay between external forces in driving the processes inherent in postdisaster recovery efforts. In addition, although past studies have illuminated the consequences of fire incidents, encompassing damage to property and supply chain disruptions (see also Peek and Guikema 2021), there has been limited attention given to how institutional deficiencies in the aftermath create conditions that accelerate business failure. The study also contributes to the burgeoning scholarly discourse on institutional dysfunction in times of crisis (Shrestha et al. 2010) by offering new insights into the nexus between institutional dysfunction and disaster events resulting in disaster‐induced business demise.

The rest of the paper unfolds as follows. In the next section, a comprehensive review of the existing literature on institutional dysfunction and business is provided. Following this, we outline the research methods and approaches to mobilize different sources of data. The penultimate section outlines the key findings pertaining to institutional dysfunction. This is then followed by the final section, which outlines a new research agenda and discusses the limitations of the approaches adopted.

## 2 Institutional Dysfunction: A Conceptual Integration

As defined by North (1990), institutions can be viewed as “the rules of the game in a society or, more formally, the humanly devised constraints that shape human interaction” (p. 3). Institutions tend to be more prevalent and influential when examining conditions for businesses in developing economies (Peng 2022; World Bank 2002). Studies indicate that formal aspects of institutions relate to rules, laws, and regulations that either facilitate or impede market functions (Peng 2022; World Bank 2002). The informal aspects of institutions are fundamentally unwritten and encompass the historical and traditional legacies that permeate and shape societies, including culture, norms, customs, and traditions (Helmke and Levitsky 2006; North 1990; Peng 2022).

One influential scholarly stream grounded in institutions is the institutional dysfunction perspective (Barnard and Mamabolo 2022; Ofori‐Dankwa and Julian 2013). Dysfunctionality can manifest in the form of inefficiencies and corruption, often hindering the development of countries (World Bank 2002). Institutional dysfunction is generally conceptualized to encompass shortcomings or inadequacies in the formal and informal “institutions that facilitate economic activity, as well as the absence of an associated set of rewards and sanctions to enforce those rules, norms, and belief systems” (Tracey and Phillips 2011, p. 31). Anchored in formal institutions are those officially established and enforced by the government and its agencies. Formal institutional dysfunction can be traced to deficiencies and inadequacies related to formal rules and regulations, such as governmental inefficiencies, regulatory compliance and enforcement issues, and poor regulatory environments (Abunyewah et al. 2023; Acquaah 2007; Amankwah‐Amoah and Hinson 2024; North 1990). Informal institutional dysfunction, stemming from unwritten norms and values, can impede and undermine trust and social legitimacy, affecting the functioning of the economy and business decisions (Helmke and Levitsky 2004, 2006; North 1990; Peng 2002, 2022). By adhering to the formal/legal “codes” of conduct and/or ingrained informal institutional practices, organizations would be better able to gain and sustain legitimacy in how they perform their functions (Barnard and Mamabolo 2022; North 1990; Peng 2002). Prior research has shown that these deficiencies can manifest in various ways, such as weak legal enforcement frameworks, incompetent government agencies and regulatory bodies, high incidences of corruption, weak intellectual property rights, government red tape, and inadequate basic infrastructure, including transportation, energy, and sanitation, that support business activities and the general economy (Amankwah‐Amoah et al. 2019; Chung and Luo 2008; Luo et al., 2009; Khanna & Palepu, 1997; Ofori‐Dankwa and Julian 2013; World Bank 2002). By recognizing the potentially pervasive influence of institutions, organizations can cultivate conditions that gain legitimacy as a means of competing and outsmarting market competition (North 1990).

A stream of research indicates that dysfunctional institutions, displaying government inefficiency, delays, bureaucratic inefficiencies, regulatory bottlenecks, corruption, and lack of transparency, have the potential to erode trust and legitimacy (see Acquaah 2011; Acquaah and Eshun 2010; Peng et al. 2023; World Bank 2002).

Research has also shown that these deficiencies can impede economic activities and affect how the market functions (see Khanna and Palepu 2005; North 1990). These deficiencies also create a permission structure that, over time, manifests to hamper the development of new ventures. For instance, the lack of or poorly enforced intellectual property rights can often create conditions that allow rampant counterfeiting of genuine brands and companies’ products and services (Amankwah‐Amoah 2023). This is particularly prevalent in developing countries, where weak legal and regulatory enforcement often hampers core pillars of market functioning, such as contract enforcement and interorganizational dispute resolution. The prevalence of high levels of corruption and lack of transparency in government decisions has the potential to erode public trust in government and government institutions (World Bank 2002), thereby leading some businesses to divert resources toward lobbying, political domination, and other political activities as a means of competing (Boso et al. 2023). Thus, addressing institutional dysfunction has the potential to create a fertile environment for business development and consequential economic growth. Thus, institutional dysfunction epitomizes the intricate interplay between formal and informal institutional conditions and their inadequacy in supporting and facilitating economic activities.

## 3 Methods and Analysis

### 3.1 Research Context: Ghana

After gaining independence in 1957 and becoming a republic in 1960, Ghana's history in the late 1960s and 1970s was marred by political instability and government overthrows, leading to economic decline, de‐industrialization, and stagnation (Aryeetey and Kanbur 2017; Jedwab and Osei 2012). By the end of the 20th century, there was an increasing recognition that Ghana had failed to fulfill its full potential and take its rightful place at the center of the global economy. The country continued to be plagued by the underdevelopment of entrepreneurial activities, high levels of poverty, and limited industrialization (Jedwab and Osei 2012). In the early 2000s, following a successful transition from one political party to another after a period of single‐party rule, the new government under J.A. Kuffour announced a new era, known as the “Golden Age of Business” (Adomako et al. 2015). This culminated in a host of political and economic reforms designed to create a more conducive atmosphere for business development and entrepreneurship (Aryeetey and Kanbur 2017). Buoyed by increasing political stability in a region with pockets of unstable countries, Ghana emerged as an emerging economy in Sub‐Saharan Africa and is often seen as “Africa's democratic poster child” (African Business 2017, p. 82).

Since the year 2000, Ghana's population has grown from 19,665,502 to 34,121,985 in 2023, thereby exerting further pressure on public services, leading to rural–urban migrations to major cities such as Kumasi, Accra, Tamale, and Tema (Worldometers 2023). Over the six decades since its independence, there has been an increasing number of fire outbreaks, which have been further exacerbated by a lack of or limited adherence to effective health and safety regulations. Since the growth and geographical concentration of shopping centers and stores in the early 2000s, there has been an increasing incidence of fire outbreaks, not only in shopping centers and complexes but also in major cities. In 2020 alone, there were at least five major fire outbreaks across the country, causing damage to properties and leading to the closure of many viable businesses, as well as the destruction of numerous shopping complexes. Reports of fire incidents in Ghana's major markets, such as Kejetia Market in Kumasi and Makola Market in Accra, and the accompanying carnage and damages from these incidents have brought them to public attention, but limited efforts have been made to curb the incidence of fire outbreaks (Amenuveve 2023; Ghanaweb 2023). Combating and mitigating fire outbreaks are characterized by the involvement of multiple vital stakeholders, including market traders and businesses, consumers, Ghana's Environmental Protection Agency, the Ghana National Fire Service, the Ghana Health Service, and the Ambulance Service (Abunyewah et al. 2023).

For instance, in the March 2023 incident, a major fire occurred in Kejetia Market in the Ashanti Regional capital after the 2021 outbreak, and in October 2023, there was another major incident in Accra (Amenuveve 2023; Ghanaweb 2023). In October 2023, over 200 shops and makeshift structures at the Makola Mall were destroyed in a fire outbreak, thereby forcing the local authorities to temporarily close down the mall (Amenuveve 2023). This led to the destruction of many products from companies, including cosmetics and jewelry. The proliferation of fire outbreaks serves as an additional impetus for scholarly examination of how fire outbreak‐induced business failure impacts entrepreneurs and their potential successive ventures.

### 3.2 Method

In light of the limited insights on the effects of institutional dysfunction in underdeveloped countries, a qualitative inductive method was considered the most appropriate to offer more robust and in‐depth insights into this complex issue (Bell et al. 2022; Gehman et al. 2018; Lim 2025; Yin 2018). Accordingly, semistructured interviews were considered the most suitable method to allow for greater flexibility and adaptability in eliciting insights from the informants (Bell et al. 2022). We adopted semistructured interviews with entrepreneurs/business owners in Ghana affected by fire incidents, whose businesses were eventually closed down after the fire outbreak in the markets. The informants were owners of retail outlets affected by major local fires. The respondents interviewed are part of a wider study into business failures in Ghana. In all, 12 affected business owners and three government officials working in the area of fire prevention were interviewed from 2020 to 2023. The interviews were recorded and transcribed within 24 h. In addition to data from the informants, we also mobilized reports and information from businesses’ annual reports, financial statements, past social media posts of some businesses, small businesses, and other trade association documents, as well as government reports and information on fire incidents. In addition, one of the researchers also visited the fire‐damaged stores and sites in Kejetia Market in Kumasi to observe the damage to vital physical infrastructure such as roads, the marketplace, and stores. This was also used as an opportunity for informal discussions with multiple traders and business owners in the area. The field observations, combined with the semi‐structured interviews, also provided the researchers with an opportunity to elicit insights into the damages sustained by existing businesses. Table 1 provides details of the different respondents/informants in the study.

**TABLE 1:** Details of respondents.

Pseudonym	Educational background	Prior industry experiences	Length of Interviews	Nature of collapsed business
MK‐1	No formal higher education, only very basic level	Prior experience in government and small‐scale gold mining	30–60 min	Market trader in Kumasi
MK‐2	HND at local technical polytechnic	Teaching as well as running his store business	40–60 min	Importer and distributor
MK‐3	No formal education and used to selling agricultural produces	Large‐scale farming	Under 30 min	Retailer
MK‐4	University degree	Worked for a mining company	30–60 min	Retailer
MK‐5	Teacher training and well‐regarded	Limited industry experience	Under 30 min	Market trader
MK‐6	Junior higher education	Prior experience in “Galamsey” (illegal small‐scale gold mining) business	Under 30 min	Market trader
MK‐7	Senior higher education	Retailer and distributor	Under 30 min	Market trader
MK‐8	Higher education, prominent figure in the local community and a serial entrepreneur in Kumasi	Retailer and distributor	Under 30 min	Retailer and distributor to other traders
MK‐9	Junior higher education	Retailer and distributor	Under 30 min	Retailer and distributor to other traders
MK‐10	Senior higher education, and years of experience in the industry	Retailer and distributor	Under 30 min	Retailer and distributor to other traders
MK‐11	Postgraduate degree	Long experience in fire service in government with proven track record and operational expertise	1 h	Retail outlet
MK‐12	Higher education	Retailer and distributor	Under 1 h	Market trader
T‐1	Technical college education	Government official—Regulator and enforcer	1 h	N/A
T‐2	Technical college education	Senior officer in the fire service	1 h	N/A
T‐3	Postgraduate degree	National fire and safety authority	Under 30 min	N/A

### 3.3 Data Analysis

To provide a deeper understanding of institutional dysfunction while maintaining a systematic approach to data analysis, we adopted the analytical method advanced and illustrated by Gioia and colleagues (see Corley & Gioia, 2011; Gioia et al. 1994; Gioia et al. 2013; Gioia, 2021). The Gioia and colleagues’ approach has been found to consistently meet rigorous scientific standards of quality (Magnani and Gioia 2023). This method involves developing first‐order, second‐order, and aggregate themes derived from semi‐structured qualitative data (Corley & Gioia, 2011; Gioia et al. 2000; Gioia et al. 2013; Gioia, 2021; Lim 2025; Magnani and Gioia 2023). As explained by Gioia and colleagues, the first‐order dimension signifies surface‐level or foundational characteristics and raw insights derived from interview data and field notes (Corley & Gioia, 2004; Gioia et al. 2013). The initial round of first‐order coding (see Corbin and Strauss 2008) integrated insights from archival documents provided by informants, interview transcripts, and other secondary data sources. Guided by the first‐order (open) coding, the second‐order (axial coding) dimensions represent a deeper analysis of the issues, identifying pivotal patterns, issues, and interconnected themes derived from the first‐order dimension (Corley & Gioia, 2011; Gioia et al. 2013). This process aimed to provide a foundational understanding of the effects of institutional dysfunction in the aftermath of fire outbreaks.

**FIGURE 1:**
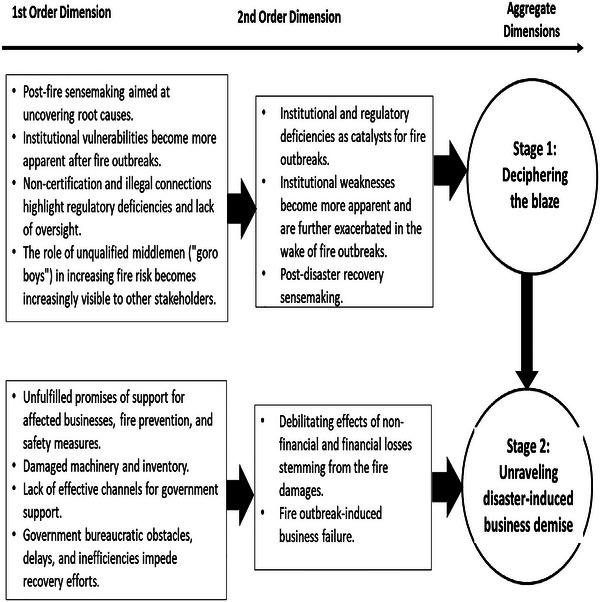
Data structure and emerging themes.

To further illustrate this process leading to the findings, two of the key themes, such as “non‐certification and illegal connections, highlight regulatory deficiencies and lack of oversight,” and “the role of unqualified middlemen (“goro boys”) in increasing fire risk becomes increasingly visible to other stakeholders,” were integrated to deduce a second‐order theme focusing on “institutional and regulatory deficiencies as catalysts for fire outbreaks” as the outcome of the sense‐making process. The aggregate dimension represents a cumulative effort that synthesizes the second‐order themes to deduce overarching themes related to institutional dysfunction in developing countries. This integrative approach is effective in uncovering deeper insights into key patterns, relationships, and overarching themes (Lim 2025; Magnani and Gioia 2023; Nag et al. 2007). Based on these themes, we identified two distinct waves in the evolution of the effects of institutional dysfunction in developing countries. Figure 1 illustrates the data structure and emerging themes, shedding light on how institutional dysfunction in the aftermath of crises and disasters acts as an accelerator, leading to business failure.

## 4 Main Findings

The analysis reveals two interconnected stages of how institutional dysfunction shapes postdisaster business recovery efforts, as demonstrated in Figure 1. Often, institutional dysfunctions, such as a lack of legal enforcement mechanisms and sanctions, coupled with weak governance systems, appear to have created conditions for limited regulatory compliance and ineffective fire risk management.

### 4.1 Stage 1: Deciphering the Blaze

Our field data analysis suggests that sensemaking and uncovering the seeds of institutional dysfunction. As the immediate postfire outbreaks come to an end, the entrepreneurs enter a new stage focusing on a deeper sense‐making geared toward uncovering the root causes. One of the visible characteristics of fire outbreaks has often been the physical infrastructure damage and destruction of organizational assets and resources, such as inventory, machinery, equipment, and general merchandise. As conveyed by Informant MK‐8, a prominent figure in the local community and a serial entrepreneur in Kumasi:

*“I was awakened at night by my son that there was a fire in the market. I rushed there… there was smoke everywhere around the market, and you could see ashes. I then knew everything was gone…”*

Another interviewee (MK‐1) expounded upon the key points alluded to:

*“I thought the fire would be tough, and then COVID happened… it became too tough for the business to survive… After the church service for the victims, I closed the business for good. Well, there was no door to close… the fire destroyed everything.”*

As the initial shock of the disaster subsides, a period of sensemaking begins, aimed at understanding the effects of institutional dysfunction. A noteworthy observation by another informant (Interviewee MK‐8) with a wealth of industry and retail experience by noting:

*“When you see the way trading is done, no one checks for health and safety inside each store. We have to check and verify wires, sockets, plugs, and appliances in every store. We do not do this, and they [authorities] claim we do not know why the fire starts… We have to do this to stop these [fire outbreaks].”*

During this phase, institutional vulnerabilities, such as a lack of effective regulations, inefficiencies in fire services organizations, and poor fire safety and prevention measures, which had operated silently in the background, became more visible and apparent in the aftermath of the outbreak. The institutional dysfunction appears to have cultivated a culture of ineffective compliance and adherence to fire and safety rules and a lack of preventative measures. As another senior fire and safety officer pointed out:

*“I guess the source of the problem is that we have a very high illiteracy rate in this country, and many people do not understand why they should invest in fire extinguishers at their business premises. Some do not even know the phone number of the fire service. Do you know in this country have people who build mansions and do not want to invest in fire extinguishers…What I have seen is that often when a fire starts, they try to fight it, and by the time they call us, the fire has already gotten out of control…this explains what is happening” (Informant T‐3)*.

Another officer in the fire service (T‐2) maintained:

*“We have so many traders who do their cooking at the market place with no proper safety measures, and this also causes some of the fires.”*

Given that the country's economy is epitomized by a high level of informality, uncontrolled growth of business infrastructure, coupled with poor environmental and safety regulations within a market space, it increases the fire hazards (Abunyewah et al. 2023; Nyame‐Asiamah et al. 2023). In the aftermath of a disaster, another informant (MK‐5), who is a widely respected owner in the retail sector, emphasized:

*“The root of the matter is clear… visit any of the markets, and you will see no respect for basic safety rules. You often see one plug for ‘hundreds’ of appliances.”*

Drawing from her prior expertise in selling products, Informant MK‐3 provided this insightful suggestion:

*“I used to take all the preventative measures to make appliances safe, store goods properly… but it takes one trader to spread the fire. Everybody should be forced to follow all rules.”*

This stage was characterized by the immediacy of uncovering that the predisaster institutional weaknesses exacerbated the conditions for businesses in the aftermath. Informant MK‐11 has a long experience in the fire service in the government with a proven track record and operational expertise. The interviewee maintained:

*“We know what is causing this, unqualified electricians, fake appliances and ‘engineers’… but they do not want to tackle the problem… listen to this, a few years ago, a fire started in the market, and residents called the fire services. The man who took the call said we do not have fuel for your cars… How can we deal with these problems when we cannot fix this basic problem.”*

Informant T‐1, a regulator and enforcer of fire regulations in the country, conveyed:

*“I have seen fires that started a few meters from the firefighters’ building. How can this happen… sometimes I hear they run out of water to quench the blaze… can you believe this?”*

Reflecting on decades of experience in the industry, one interviewee (MK‐8) attested:

*“It is clear to me that the (GNFS) Ghana National Fire Service is incompetent, not efficient and fails to invest in prevention.”*

It was observed that the weak and poor enforcement of fire safety regulations, product safety standards, and building codes has the potential to lead to fire outbreaks.

#### 4.1.1 The Use of Unqualified Middlemen

The empirical evidence gleaned from field data analysis clearly indicates that in the absence of effective enforcement and punitive punishment for uncertified electricians and other technical personnel, health and safety standards are often compromised. Indeed, certified businesses and professional individuals are expected to adhere to high safety protocols and knowledge, which goes a long way in preventing fire outbreaks. Nevertheless, there is also noncertification and rampant operation of numerous middlemen, known as “Goro boys” (i.e., largely unqualified and uncertified workers), often without formal and high‐level education appears to have played a pivotal role in creating the conditions that lead to fire outbreaks. Another Informant (MK‐10), known for years of experience in the industry, remarked:

*“After every one of these fires… you see the damage and lives ruined, but it keeps happening. No one tackles the problem. We have too many unqualified people doing wiring and fixing electrical appliances… businesses around this market must be forced to use only professionals, period.”*

In artfully articulating this perspective, a respondent (MK‐2) highlighted the reality of the situation in the country that has manifested this:

*“You will find that building works, electrical services, and even passport services are now provided by ‘goro boys'… If we do not compel people to only use professionals for this work, we will continue to witness fires in this market.’”*

Another finding, anchored in the field data, indicates that this problem has been further compounded by the problem of rampant illegal connections to the electricity and national power grid, thereby increasing the risk of fire not only in market centers but also in residential areas. In essence, Ghana also lacks any effective schemes for registering, monitoring, and deregistering business activities. This insight was also offered by Informant (MK‐4):

*“All the electrical and appliance works are being done by ‘goro boys’. They are building new stores, fixing electrical problems, and repairing the roof. They claim they can do everything, but they often leave, and then there are fire outbreaks. We need to require that these works are done by professionals who are certified by someone. The use of ‘goro boys’ is the root of the problem.”*

During the course of the interview, a respondent offered this perspective, stating:

*“I am not an electrician, but I can see the quality of work some are doing. When you visit other stores, you can observe the issues and sub‐standard materials being used” (MK‐6)*.

By bypassing security and safety standards, many small businesses are able to reduce the cost of their operations while concurrently increasing the risk of fire outbreaks. The incidence of noncertification of technical personnel can be traced to institutional voids in the form of regulatory deficiencies, inadequate oversight, and a lack of effective regulatory enforcement.

### 4.2 Stage 2: Unraveling Disaster‐Induced Business Demise

Our field data indicates that Stage 2 was exemplified by multiple promises made by local and national political actors immediately after the disaster, culminating in business failure induced by the outbreak. In the aftermath of the outbreaks, political actors often pledged support for affected businesses, investment in fire prevention measures, and safety measures. Another informant conveyed:

*“Yes, I took some time to walk around the affected areas. There were ashes and ashes. It was emotional for me. Shops after shops burned down. After all the promises, we are still waiting, and hope rises from the ashes” (MK‐9)*.

Our data indicates that bureaucratic bottlenecks and extended delays in obtaining institutional support not only exacerbate the financial stress of the business but also hinder its revival, access to credit, and access to resources. The quote below illustrates the perspective of one entrepreneur and trader in one of the affected markets who articulated:

*“That is why I had to close the business… I lost so much money through the fire, and then the promise from XXX [an important political figure] was not fulfilled. We got nothing from them. I could not continue paying the workers and had to close down for good” (MK‐10)*.

Often, the numerous promises of government officials fail to materialize, prolonging the agony of business owners. As one regulator, informant T‐2a, maintained:

*“From where I stand, I can see it is always like a ‘never‐ending nightmare’ for the affected businesses, and they struggle to get any help.”*

Another noteworthy observation by the informants was reflected in the following statement:

*“The business survived the first fire, with destroyed merchandise, but the second fire, after a few years, was too damaging, so I had to close and start something different… When you do not have any insurance, then you have to pray for help. At the moment, I am looking for a visa to go to America” (MK‐7)*.

According to Thebftonline.com (2023), three out of four shops in the recently inferno‐affected area of the Kejetia Market in Kumasi (i.e., Ghana's second‐largest city) do not have any form of insurance and view insurance companies as dishonest. Indeed, the market consists of numerous traders and informal businesses that are increasingly affected by infernos, often destroying many shops and businesses in the area. Following the corrosive effects of institutional dysfunction in the aftermath of the disaster, failure becomes imminent. In the weeks after the outbreak, existing customers of the businesses began to switch to alternative firms and change suppliers. This termination of relationships and loss of current customers create harsh conditions that eventually lead to the demise of the businesses. Often, the cost of repairing the damaged structure, restocking, and fixing infrastructure absorbs all the available cash, resulting in closure. As further elaborated by the informant:

*“We were not able to re‐start after the fire, so our regular customers left us …we were not providing them the products, so they all started looking elsewhere. Our cash reverse was not enough to keep the business open” (Informant MK‐12)*.

Coupled with the lack of or breakdown in channels to government support, characterized by multiple bureaucratic obstacles, delays, and inefficiencies, the closure of businesses becomes imminent. The failed immediate postdisaster business recovery efforts culminate in a downward spiral toward exit. Accompanying fire outbreaks have often resulted in damaged machinery and inventory, leading to financial losses and the cessation of operations. In his closing advice, one informant within the national fire and safety authority added:

*“We need to better educate people to avoid overloading sockets…follow fire safety measures at all shops in the marketplace, at home, and everywhere.” (Informant T‐3)*.

## 5 Discussion and Implications

This research aimed to provide insights into how institutional dysfunction shapes postdisaster recovery. Utilizing insights from businesses affected by marketplace fire outbreaks in a developing economy, this study offers a two‐stage explanation of how institutional dysfunction manifests. The research observed that institutional dysfunction unfolds in two stages, capturing the postdisaster business recovery responses. It highlights the sensemaking process following the fire outbreaks, focusing on institutional shortcomings, inadequacies in formal institutional support, and a lack of preventative measures as primary causes (Stage 1). This is followed by the potent manifestation of failed institutional support in the aftermath of the fire incidents, such as unfulfilled government promises of support, lack of insurance, and a shortage of cash reserves to support the businesses, ultimately leading to disaster‐induced business demise (Stage 2). In essence, the aftermath of fire outbreaks reveals the fragilities of existing formal and informal institutions that impede business activities. These outbreaks act as accelerators in uncovering weaknesses in existing institutional support for business activities, as well as revealing the absence of slack resources, insurance, record‐keeping, and proper documentation typical of small business operations, which hinder postoutbreak effects and exacerbate conditions that lead to business failure. The evidentiary weight of the findings supports the assertion that institutional dysfunction is seen as an accelerator in the postfire outbreak period, resulting in the depth of dysfunction, its effects, and multiple business failures. Thus, the two stages elucidate the cascading effects of institutional dysfunction on postdisaster business recovery.

### 5.1 Theoretical Contributions

From a theoretical standpoint, although past studies have examined the effects of disasters (Mostafavi and Ganapati 2021; Peek and Guikema 2021; Sherman‐Morris et al. 2021), prior research has failed to account for how fire outbreak‐induced business failure manifests. In line with the prevailing institutional dysfunctional literature (Khanna & Palepu, 1997; Luo et al., 2009; Ofori‐Dankwa and Julian 2013; Peng 2022), this study illuminates and addresses this gap by capitalizing on insights from developing countries to shed light on how institutional dysfunction in the wake of disasters can create the conditions leading to business failure. Building upon the institutional dysfunction literature presented previously (North 1990; Scott 2013), this study addresses this deficiency in the current literature by developing and illustrating a phase model that explains the processes, decision points, and effects of fire outbreaks.

Departing from conventional wisdom, the study illuminates the destabilizing influences of fire outbreaks and institutional dysfunction as accelerators in creating conditions in the postcrisis period. By delving into the interplay between depleted or lost organizational resources in the context of institutional dysfunction, we shed light on the accelerated process. While business failure has garnered significant and growing scholarly attention (Contreras et al. 2023), a review of the literature indicates that the effects of disasters manifest in shaping the closure process remain largely unaccounted for in the current literature. Drawing on data from failed enterprises in Ghana, this investigation contributes to the current literature by conceptualizing business failure as an outcome of externally driven conditions, shedding light on the intersection between disaster studies and business failure. Given that around 80% of Ghana's workforce operates in the informal sector, typified by informal institutions (Ghana Statistical Service 2013), the effects of institutional dysfunctions are likely to differ fundamentally from conditions in advanced economies. For instance, a typical Ghanaian marketplace tends to be heavily congested with numerous informal traders and their customers (Abunyewah et al. 2023; Boamah et al. 2020). Accordingly, this study addresses the current paucity of scholarly works on how the effects of informal institutions manifest in hampering disaster recovery efforts.

### 5.2 Practical Contributions

The analysis and insights derived offer several noteworthy practical implications. First and foremost, addressing the fire outbreak issue necessitates multi‐stakeholder collaboration, including the Ghana Fire Services, businesses, and local authorities, toward creating and implementing stringent regulatory and certification protocols. These protocols would go a long way in establishing a more concrete culture of elevating safety, regulatory oversight, and compliance. The study indicates a general need for upgrading existing infrastructure and national policies directed toward combating the root causes of fire outbreaks, such as faulty appliances. Furthermore, there is a need for a national training program geared toward market traders, businesses, and their workers, equipping them with basic skills and expertise to mitigate the potential risks of fire outbreaks. There is also a need to create a new national culture of fire education and training geared toward businesses to update their skills and expertise in fire prevention in all major business areas. Such an approach has the potential to help not only businesses in cities but also those in rural areas as a means of protecting their assets.

Additionally, there is a role for the markets in providing comprehensive fire insurance policies, and businesses should also be obligated to obtain such insurance as part and parcel of their licenses to operate in certain areas. In addition, the analysis indicates the need for the government to legislate, forcing businesses in market areas to invest in advanced fire detection technologies such as fire alarms, automated fire suppression systems, and warning systems linked to government fire agencies as a means of detecting, responding to, and combating fire incidents.

### 5.3 Limitations and Future Research Directions

In spite of valuable practical implications, there are several limitations of the study linked to the directions for future research. First, given that the sample was drawn from a developing economy, the applicability of these findings to other countries is limited. Another possible limitation concerns the limited sample size of 12 affected business owners and three government officials, and its event‐specific nature. Future research could further enrich this study by encompassing much larger samples from both developed and developing economies. The study also mainly focuses on the perspectives of founders/owners and their lived experiences, which demonstrates a stronger reflection of their biases and feelings. Another promising domain for future research would be an examination of the long‐term effects of institutional dynamics that lead to such fire outbreaks in different contexts and industries in the Economic Community of West African States (ECOWAS), such as Côte d'Ivoire, The Gambia, Mali, Guinea, Senegal, and Guinea‐Bissau. In view of the differential effect of the outbreak on different types of businesses, there is a need for future research to focus on large firms, such as multinationals and state‐owned enterprises. By addressing the gaps pertaining to the effects of institutional dysfunction in the current literature, it is hoped that this research serves as a catalyst for new research on business failures induced by natural disasters, which have become increasingly common in the wake of climate change.

### 5.4 Conclusion

In closing, this field study advances the scholarly discourse on risk analysis (Gokmenoglu and Dasci Sonmez 2024; Nyame‐Asiamah et al. 2023) and recovery from disasters (Xie et al. 2018) by examining the effects of institutional dysfunction on postdisaster recovery. This study's focus on developing countries, shaped by institutional deficiencies, provides valuable insights into the different types of risks that manifest in the aftermath of fire outbreaks. It is hoped that this research serves as a catalyst for new streams of inquiry into how institutional dysfunction contributes to fire outbreaks in sub‐Saharan African countries and beyond.
